# MicroRNA Expression Profiling in Age-Dependent Renal Impairment

**DOI:** 10.3389/fmed.2022.849075

**Published:** 2022-05-13

**Authors:** Katsunori Yanai, Shohei Kaneko, Hiroki Ishii, Akinori Aomatsu, Keiji Hirai, Susumu Ookawara, Yoshiyuki Morishita

**Affiliations:** ^1^Division of Nephrology, First Department of Integrated Medicine, Saitama Medical Center, Jichi Medical University, Saitama, Japan; ^2^Division of Intensive Care Unit, First Department of Integrated Medicine, Saitama Medical Center, Jichi Medical University, Saitama, Japan

**Keywords:** MicroRNA, age-dependent renal impairment, unilateral ureteral obstruction, adriamycin, SAMP1, messenger RNA, kidney

## Abstract

**Background:**

Age-dependent renal impairment contributes to renal dysfunction in both the general population and young and middle-aged patients with renal diseases. Pathological changes in age-dependent renal impairment include glomerulosclerosis and tubulointerstitial fibrosis. The molecules involved in age-dependent renal impairment are not fully elucidated. MicroRNA (miRNA) species were reported to modulate various renal diseases, but the miRNA species involved in age-dependent renal impairment are unclear. Here, we investigated miRNAs in age-dependent renal impairment, and we evaluated their potential as biomarkers and therapeutic targets.

**Methods:**

We conducted an initial microarray profiling analysis to screen miRNAs whose expression levels changed in kidneys of senescence-accelerated resistant (SAMR1)-10-week-old (wk) mice and SAMR1-50wk mice and senescence-accelerated prone (SAMP1)-10wk mice and SAMP1-50wk mice. We then evaluated the expressions of differentially expressed miRNAs in serum from 13 older patients (>65 years old) with age-dependent renal impairment (estimated glomerular filtration ratio <60 mL/min/1.73 m^2^) by a quantitative real-time polymerase chain reaction (qRT-PCR) and compared the expressions with those of age-matched subjects with normal renal function. We also administered miRNA mimics or inhibitors (5 nmol) with a non-viral vector (polyethylenimine nanoparticles: PEI-NPs) to SAMP1-20wk mice to investigate the therapeutic effects.

**Results:**

The qRT-PCR revealed a specific miRNA (miRNA-503-5p) whose level was significantly changed in SAMP1-50wk mouse kidneys in comparison to the controls. The expression level of miRNA-503-5p was upregulated in the serum of the 13 patients with age-dependent renal impairment compared to the age-matched subjects with normal renal function. The administration of a miRNA-503-5p-inhibitor with PEI-NPs decreased the miRNA-503-5p expression levels, resulting in the inhibition of renal fibrosis in mice via an inhibition of a pro-fibrotic signaling pathway and a suppression of glomerulosclerosis in mice by inhibiting intrinsic signaling pathways.

**Conclusion:**

The serum levels of miRNA-503-5p were decreased in patients with age-dependent renal impairment. However, inhibition of miRNA-503-5p had no effect on age-dependent renal impairment, although inhibition of miRNA-503-5p had therapeutic effects on renal fibrosis and glomerulosclerosis in an *in vivo* animal model. These results indicate that miRNA-503-5p might be related to age-dependent renal impairment.

## Introduction

Age-dependent renal impairment is one of the most common diseases of end-stage renal disease (1). Although the pathogenesis of age-dependent renal impairment is not completely cleared, a lot of genetic and environmental factors such as genetic susceptibility, smoking, salt intake, exposure of lead, thrombophilia, metabolic syndrome, and low birth weight have been reported to be associated with the progression of age-dependent renal impairment (2). It was also reported aging-related arterial intimal fibrosis causes ischemia in the aging kidneys, which first causes glomerulosclerosis, resulting in contributing to the development of age-dependent renal impairment (3). The tubulointerstitial changes could also be derived from the age-associated arteriosclerosis and consequent hypoxia/ischemia in both the medulla and cortex of the kidney, which leads to the development of age-dependent renal impairment (3). The exact molecular mechanisms underlying age-dependent renal impairment have not been established.

MicroRNA (miRNA) is a non-coding, single-stranded RNA molecules that are 20–25 nucleotides in length. They have the function of regulating gene expression at a post-transcriptional level via nucleotide base pairing between the complementary sequence of miRNA and the 3'-untranslated regions (3' UTRs) of messenger RNA (mRNA) (4). MiRNAs are reported to play pivotal parts in progression of inflammation, cancer, and many other pathological and physiological conditions (5). The relation between miRNA and age-dependent renal impairment is not yet understood.

We have observed no published reports about the relationship between miRNA and age-dependent renal impairment. The possibility of applying miRNA as a biomarker and therapeutic target for age-dependent renal impairment has apparently not been investigated. Therefore, we conducted screening using animal models to identify miRNAs that may be involved in age-dependent renal impairment. Then, we evaluated the miRNAs selected in the screening for use as biomarkers in older patients with age-dependent renal impairment. Also, we evaluated the therapeutic effect of a miRNA inhibitor on the inhibition of age-dependent renal impairment in an animal model *in vivo*. Since normal strains of mice rarely exhibit significant age-dependent renal impairment, we used senescence-associated mouse prone (SAMP1) mice, which have been confirmed to undergo age-dependent renal impairment; we used senescence-associated mouse resistant (SAMR1) mice, which undergo normal aging, as a control.

## Methods and Materials

### Ethical Considerations

This research was advanced in accord with the Declaration of Helsinki and admitted by Jichi Medical University Ethics Committee. The experimental protocol was admitted by Jichi Medical University Animal Ethics Committee and was conducted according to Jichi Medical University Experimental Animal Committee's guide on the use and care of laboratory animals. All patients provided informed consent.

### Mouse Model of Age-Dependent Renal Impairment

We purchased male SAMP1 10-week-old age (wk) mice and male SAMR1 10-wk-old mice that weigh 25–30 g from CLEA Japan (Tokyo). We housed them under antibody-free micro-isolator antiviral conditions and fed normal chow until the age of 50 weeks.

### Mouse Model of Renal Fibrosis

We purchased 8-week-old C57BL/6 male mice that weigh 20–25 g from CLEA Japan. We housed them under antibody-free micro-isolator antiviral condition. To cause renal fibrosis, unilateral ureteral obstruction (UUO) was created as a following report (6). We obstructed the ureter completely by double ligating with 4-0 silk sutures.

### Mouse Model of Glomerulosclerosis

We purchased 8-week-old BALB/c male mice that weigh 20–25 g from CLEA Japan and housed them under antibody-free micro-isolator antiviral conditions. For the induction of glomerulosclerosis, 10 mg/kg adriamycin was intravenously injected as described (7).

### miRNA-503-5p

miRNA-503-5p and control-miRNA were purchased from GeneDesign (Osaka, Japan). The sequences are as follows: miRNA-503-5p were 5′-UAGCAGCGGGAACAGUUCUGCAG-3′ (sense) and 5′-GGGGUAUUGUUUCCGCUGCCA-3′ (antisense), and the sequences of the control-miRNA were as follows: 5′-UUCUCCGAACGUGUCACGUTT-3′ (sense) and 5′-ACGUGACACGUUCGGAGAATT-3′ (antisense).

### Preparation of miRNA-503-5p-Inhibitor-PEI-NPs and Control-miRNA-PEI-NPs

We purchased linear polyethylenimine nanoparticles (PEI-NPs) (*in vivo*-jet PEI®) from Polyplus-Transfection SA (Strasbourg, France) and mixed them with 5% glucose solution at a concentration of 50 μM. Then, we incubated them for 15 min at 15°-25°C. We intravenously injected a total of 200 μL of miRNA-PEI-NPs (miRNA: 5 nmol, the ratio of nitrogen [N] in polymer to phosphate [P] in nucleic acid = 6) into each mouse from the tail vein. We created control-miRNA-PEI-NPs in the same way.

### Delivery of miRNA-PEI-NPs to the Kidneys of SAMP1 Mice

miRNA-503-5p-inhibitor-PEI-NPs were administered intravenously to each mouse nine times once a week compared with SAMP1-20wk (SAMP1-20wk+miRNA-503-5p-inhibitor-PEI-NP). The following two groups acted as controls: SAMP1-20wk mice with no injection (SAMP1-20wk + no injection), SAMP1-20wk mice with control-miRNA-PEI-NPs (SAMP1-20wk + control-miRNA-PEI-NP). We euthanized the mice 10 weeks later at 30 wk. The kidneys were then removed and washed in phosphate-buffered saline (PBS) by reflux flow.

### Delivery of miRNA-PEI-NPs to the Kidney in UUO Mice

miRNA-503-5p-inhibitor-PEI-NPs was administered intravenously into each mouse three times, on days −1, 1, and 3 compared to UUO operation (UUO+miRNA-503-5p-inhibitor-PEI-NP). The following three groups acted as controls: sham-surgery mice (sham-surgery), UUO mice with non-injection (UUO + non-injection), and UUO mice injected with control-miRNA-PEI-NPs (UUO + control-miRNA-PEI-NP). We sacrificed the mice 8 days after UUO, and we removed the kidneys and washed them with PBS by reflux flow.

### Delivery of miRNA-PEI-NPs to the Kidney in Adriamycin-Induced Glomerulosclerosis Mice

First, we administered intravenously miRNA-503-5p-inhibitor-PEI-NPs into each mouse five times, on days −1, 1, 4, 7, and 10 in relation to adriamycin injection (adriamycin+miRNA-503-5p-inhibitor-PEI-NP). The following three groups acted as controls: mock mice (mock), adriamycin mice with non-injection (adriamycin + non-injection), and adriamycin mice administered with control-miRNA-PEI-NPs (adriamycin + control-miRNA-PEI-NP). We sacrificed the mice 14 days after the adriamycin injection, and we removed their kidneys and washed them with PBS by reflux flow.

### Microarray Analysis

We asked Hokkaido System Science (Hokkaido, Japan) to perform miRNA expression analysis with miRNA Complete Labeling Reagent and Hyb Kit (Agilent Technologies, Santa Clara, CA) in line with the manufacturer's protocols. First, 100 ng of total RNAs were dephosphorylated at 37°C for 30 min and denatured with 100% dimethyl sulfoxide at 100°C for 7 min. The specimens were then chilled on ice for 2 min. The specimens were next labeled with cytidine-5'-phosphate-3'-(6-aminohexyl) phosphate, and Cy3 (pCp-Cy3) with T4 ligase through incubation at 16°C for 2 h. After that, the specimens were hybridized on an 8 × 60K SurePrint G3 mouse miRNA array (Agilent Technologies). The arrays were gently washed with Gene Expression Wash Buffer (Agilent Technologies) in line with the manufacturer's protocols and analyzed at a resolution of 3 μm with an Agilent Technologies Microarray Scanner. Data were obtained by Agilent Feature Extraction software, ver. 12.0.3.1.

### The Process of miRNA Microarray Data and Statistical Analysis

**Figure 1:**
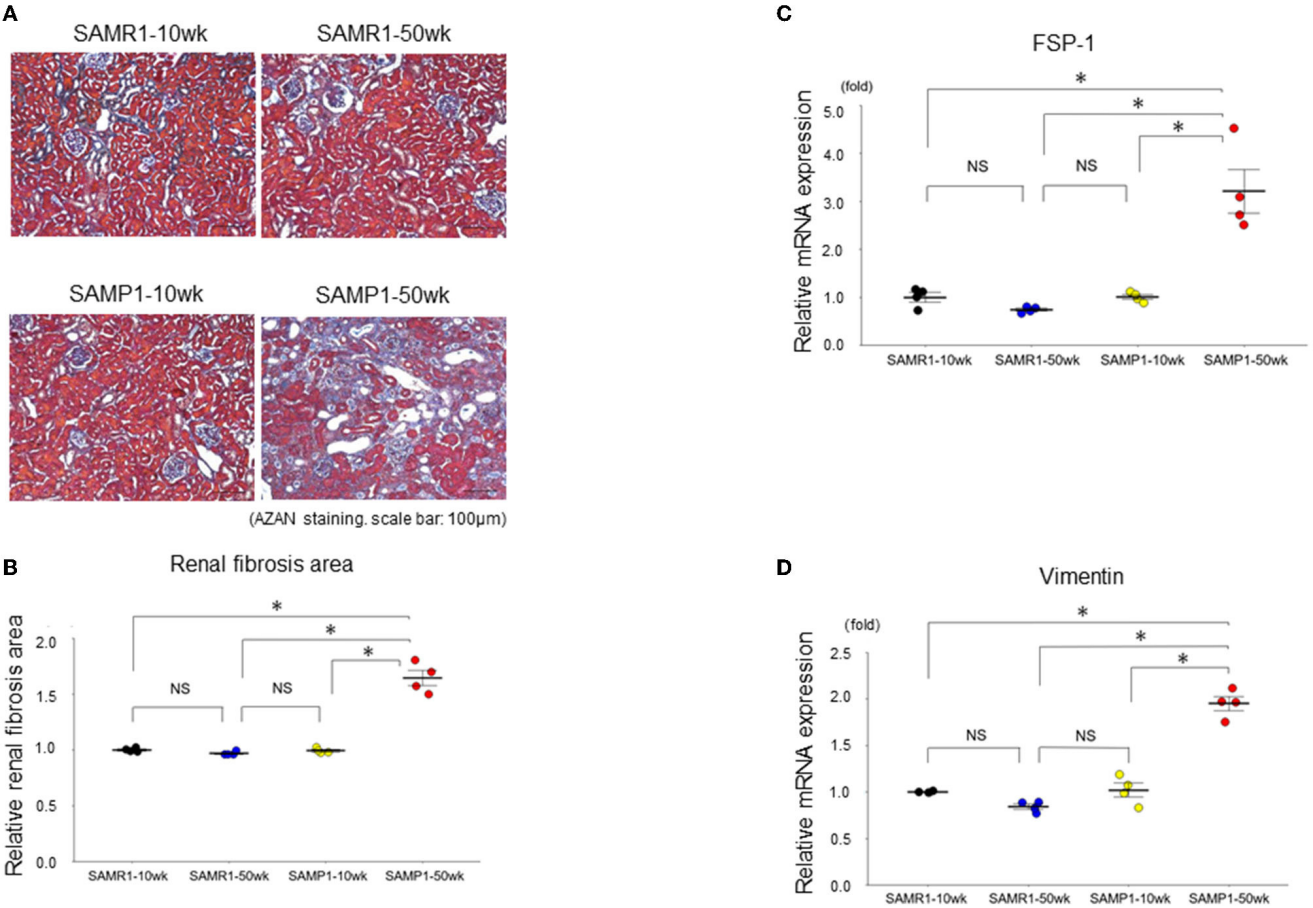
Changes of interstitial nephropathy in SAMR1-10wk mice, SAMR1-50wk mice, SAMP1-10wk mice, and SAMP1-50wk mice. **(A)** Azan staining paraffin sections. Magnification: ×200. Scale bar: 100 μm. Marked renal fibrosis was observed in SAMP1-50wk mice. **(B)** Changes in renal fibrosis area with SAMR1-10wk mice, SAMP1-10wk mice, SAMR1-50wk mice, and SAMP1-50wk mice. **(C)** qRT-PCR analysis of FSP-1 expression in each group (*n* = 4). **(D)** qRT-PCR analysis of vimentin expression in each group (*n* = 4). Values represent mean ± SE (error bars). *p< 0.05, ANOVA, Tukey's test. FSP-1, fibroblast specific-protein-1.

The Agilent data were taken into GeneSpring GX (Agilent Technologies), normalized to the 90th percentile per chip, and baseline correction was performed based on the data of the SAMR1-10wk mice (**Tables 1A–C** and Figures 1A–C). A two-way analysis of variance (ANOVA) was used to investigate statistical differences among the groups. Tukey's test was used for the *post-hoc* analysis. Differences with a probability (p)-value < 0.05 were thought to be statistically significant (8). A cluster and heat map of miRNAs that meet the conditions are shown for all the microarray analysis carried out.

### Quantitative Real-Time Reverse-Transcription Polymerase Chain Reaction

The quantitative real-time reverse-transcription polymerase chain reaction (qRT-PCR) have been explained in detail (9). Briefly, we homogenized kidney specimens with a silica homogenizer and a filter column shredder (QIAshredder column; Qiagen, Valencia, CA). We extracted mRNA from each kidney with an RNeasy Mini Kit (RNeasy®, Qiagen). Then, we reverse transcribed 1 μg of each extracted RNA with the Life Technologies Super-Script® III First-Strand Synthesis System (Thermo Fisher Scientific). We carried out the real-time qRT-PCR with Life Technologies SYBR® GreenER™ qPCR SuperMix (Thermo Fisher Scientific). We purchased primers for mouse β-actin, fibroblast-specific protein-1 (FSP-1), collagen 1A2, alpha-smooth muscle actin (α-SMA), small mothers against decapentaplegic (Smad) 2, Smad3, Smad7, podocin, desmin, B-cell lymphoma-2 (BCL-2), Bax, caspase 2, and caspase 3 from Takara Bio (Otsu, Shiga, Japan).

The primers used were as follows: collagen 1A2, α-SMA, FSP-1, vimentin, TGF-β_1_, Smad2, Smad3, Smad7, β-actin, podocin, desmin, BCL-2, Bax, caspase 2, and caspase 3. We normalized the expression level of each mRNA in relation to that of β-actin. To evaluate the expression level of miRNA, we extracted homogenized kidney solutions with a miRNeasy Mini Kit (Qiagen), and reverse transcribed 1 μg of each extracted RNA with the miScript® II RT Kit (Qiagen). We performed the real-time qRT-PCR with a miScript SYBR Green PCR Kit (Qiagen). We purchased the primers for miRNA-503-5p and U6 small nuclear RNA-2 (RNU6-2) from Qiagen. We normalized the expression level of each miRNA compared to that of RNU6-2. The resulting data are represented in relation to the sham-surgery group or mock group.

For the evaluation of the levels of miRNA expression in human serum, total RNAs from 300 mL of serum was extracted with a NucleoSpin miRNA Plasma column (Takara Bio). We reverse transcribed the isolated RNA with the miScript II RT Kit (Qiagen). We carried out the qRT-PCR with a miScript SYBR Green PCR Kit (Qiagen), and purchased the primers for miRNA-16, miRNA-142a-3p, miRNA-142a-5p, miRNA-144-3p, miRNA-155-5p, miRNA-181d-5p, miRNA-205-5p, miRNA-342-3p, miRNA-451a, miRNA-503-5p, miRNA-802-5p, miRNA-1231-5p, let-7d-3p, and miRNA-423-5p from Qiagen. We normalized miRNA expression for miRNA-16 as an endogenous control following the report in the past (10). The results are expressed as relative quantities in relation to those of an older patient (>65 years old) with normal renal function.

The expression level of miRNA-16 in healthy subjects showed high variation; we therefore used miRNA-423-5p as the endogenous control (11) when comparing the expressions of miRNA-142a-3p, miRNA-142a-5p, miRNA-144-3p, miRNA-155-5p, miRNA-503-5p, and miRNA-1231-5p between the older patients with age-dependent renal impairment and the healthy subjects. The data are expressed as relative quantities compared to the averages of the healthy subjects.

### Histological Analysis

After reflux flow with PBS, we removed the kidney and fixed them with 4% paraformaldehyde. Then, we embedded them in paraffin, sectioned, and subjected to azan staining for evaluation of fibrous changes and subjected to periodic acid-Schiff (PAS) staining for the determination of the glomerulosclerosis index. We evaluated quantitatively the degree of fibrosis by examining ten fields per section positively stained with azan at a magnification of 200-fold. We chosed randomly fields for analysis, and quantified the azan-stained areas with the image analysis software provided with the fluorescence microscope (BZ-X710, Keyence, Osaka, Japan). We evaluated quantitatively the degree of sclerosis was by examining 50 glomeruli positively stained for PAS at 200-fold magnification (12). We examined the glomerulosclerosis index as 1 point = no change in glomerulus, 2 points = proliferation of mesangial cells, 3 points = segmental sclerosis, and 4 points = total glomerulosclerosis (12).

### Serum Sample

We studied 13 older patients (>65 years old, 12 males and 1 female aged 69.4 ± 4.07 yrs) with age-dependent renal impairment by performing a qRT-PCR and comparing the patient's results with those of age-matched subjects with normal renal function (*n* = 13) examined at Jichi Saitama Medical Center, Japan between 2018 and 2021. We purchased the control subjects' serum from Cosmo Bio Company (Tokyo) and had the blood analysis for this group conducted at a clinical chemistry laboratory (SRL, Tokyo). The controls were 13 males, aged 69.6 ± 4.48 years old.

The subjects' characteristics are summarized in **Table 2A**. Age-dependent renal impairment was diagnosed based on blood and urine tests (estimated glomerular filtration ratio [eGFR] <60 mL/min/1.73 m^2^), and the results of both the urinary protein and occult blood test were negative (13). The eGFR of all the subjects was obtained by a standard equation (14). The case of one kidney was excluded, and patients with other coexisting renal disease were excluded. All patients provided informed consent.

### Statistical Analysis

We used a propensity score matching analysis to select control patients with similar baseline characteristics. Age and sex were used in the propensity model as independent variables. We performed one-to-one pair matching by identifying the control patient with a caliper width of 0.25 standard deviation and the closest log-transformed propensity score. Data are expressed as the mean ± SE (standard error). The means of the data of the healthy subjects and those of the patients with age-dependent renal impairment were compared using Student's *t*-test. We used an analysis of variance (ANOVA) for multiple comparison analysis testing among groups. If we detected statistical significance by the ANOVA, we carried out Tukey's test to compare the means of two groups as a *post-hoc* analysis. We analyzed the relation between continuous variables with Pearson's correlation test or a linear regression analysis. We used JMP software (ver. 13; SAS, Cary, NC) for all statistical analyses. Differences with a *p* < 0.05 were thought to be significant.

## Results

### Renal Fibrosis and Glomerulosclerosis in the SAMP1-50wk Mice

**Figure 2:**
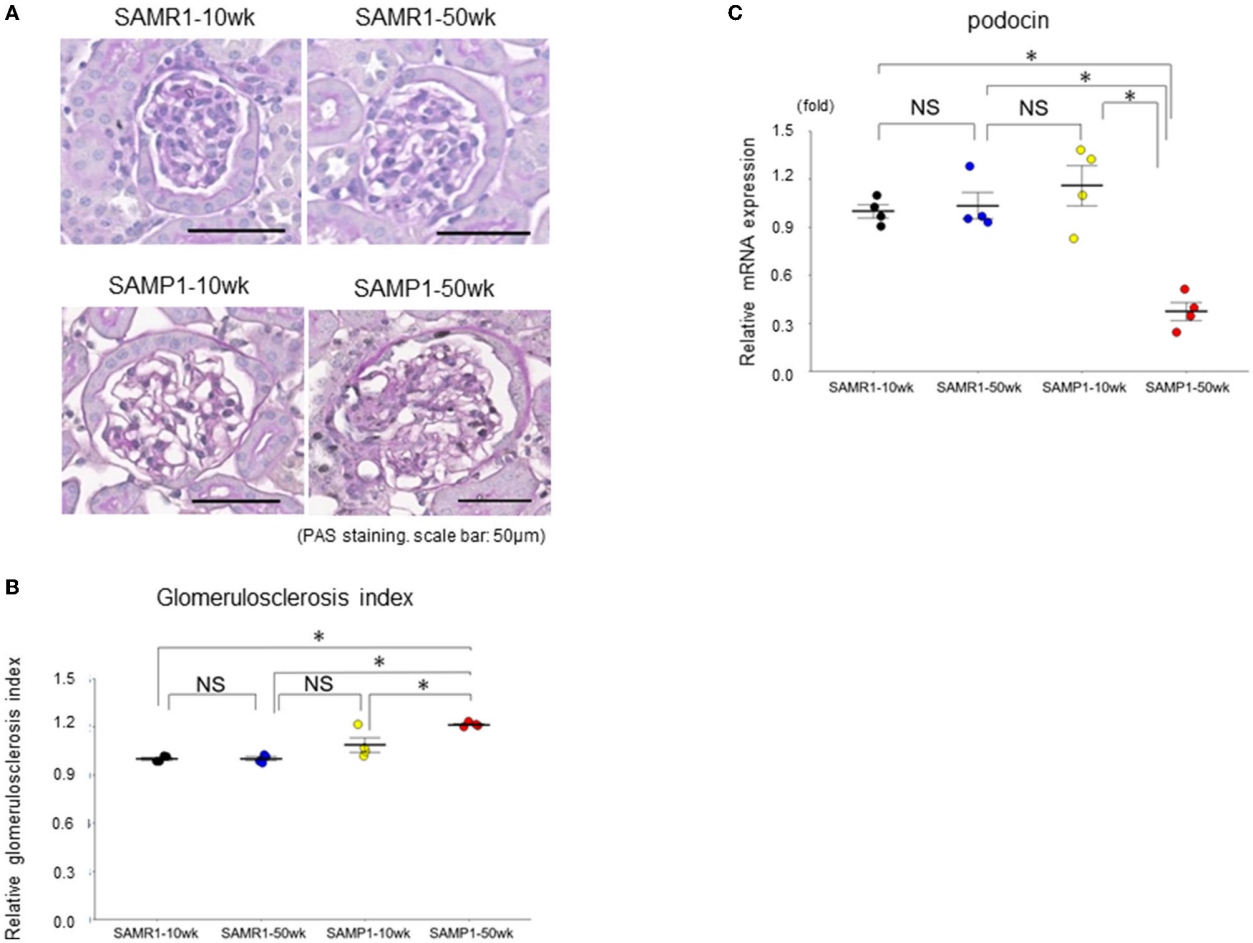
Changes of glomerulosclerosis in SAMR1-10wk mice, SAMR1-50wk mice, SAMP1-10wk mice, and SAMP1-50wk mice. **(A)** Periodic acid-Schiff-stained paraffin sections. Magnification: ×200. Scale bar: 50 μm. Marked glomerulosclerosis was observed in the SAMP1-50wk mice. **(B)** Changes in the glomerulosclerosis index of the four groups of mice. **(C)** qRT-PCR analysis of podocin expression in each group (*n* = 4). Values represent mean ± SE (error bars). *p < 0.05, ANOVA, Tukey's test.

Histologically, SAMP1-50wk mice demonstrated typical features of fibrosis and glomerulosclerosis compared to the SAMR1-10wk mice, SAMR1-50wk mice, SAMP1-10wk mice (Figures 1, 2). The qRT-PCR analysis confirmed further increases in the renal fibrosis markers FSP-1 and vimentin in the SAMP1-50wk mice (Figures 1C,D), and that the expression of the glomerulosclerosis marker podocin was decreased in the SAMP1-50wk mice (Figure 2C).

### Microarray miRNA Profiling

We screened for changes in miRNA expressions in age-dependent renal impairment with a microarray platform covering 1,881 mouse miRNAs (miRBase database release 21; www.mirbase.org). The screening results can be found using the following link: https://www.ncbi.nlm.nih.gov/geo/query/acc.cgi?acc=GSE198741. The screening revealed that 11 of the 1,881 miRNAs were significantly different in expression between pairs of SAMP1 and SAMR1 groups by two-way ANOVA: miRNA-142a-5p,−181d-5p,−205-5p,−342-3p,−467a-3p,−503-5p,−802-5p,−1231-5p,−3091-5p,−3095-3p, and−6981-5p (Table 1A). The screening also showed that one of the 1,881 miRNAs (miRNA-802-5p) was significantly different in expression between 10-wk and 50-wk groups by two-way ANOVA (Table 1B). In addition, six of the 1,881 miRNAs made a significant difference in expression among the four groups (SAMR1-10wk, SAMR1-50wk, SAMP1-10wk, and SAMP1-50wk) by two-way ANOVA: miRNA-142a-3p,−142a-5p,−144-3p,−155-5p,−451a, and let-7d-3p as depicted in (Table 1C).

**Table 1A:** miRNAs whose expressions were significantly higher (by >2.0 times) in SAMP1 mouse kidneys compared to SAMR1 mouse kidneys by microarray (SAMR1-10wk + SAMR1-50wk vs. SAMP1-10wk + SAMP1-50wk).

**MicroRNA**	**Sequence**	**SAMR1-50wk**	**SAMP1-10wk**	**SAMP1-50wk**	**Fold change (SAMP1 mice/SAMR1 mice)**	***p***
miRNA-503-5p	CTGCAGTACTGTTCCC	0.19	3.22	2.34	6.25*	<0.05
miRNA-802-5p	AAGGATGAATCTTTGTTACTGA	17.0	16.4	18.0	4.16*	<0.05
miRNA-142a-5p	AGTAGTGCTTTCTACTTTA	1.04	1.26	3.44	2.04*	<0.05
miRNA-6981-5p	GCCTTCAGCCTCTTC	0.38	9.00	5.73	11.69*	<0.05
miRNA-1231-5p	TCTCTCCTGCAGCTCT	0.99	5.25	14.5	8.76*	<0.05
miRNA-467a-3p	TGTAGGTGTGTGTATGTATA	1.02	2.95	8.05	4.83*	<0.05
miRNA-342-3p	ACGGGTGCGATTTCTGT	1.02	32.1	61.7	44.15*	<0.05
miRNA-205-5p	CAGACTCCGGTGGAAT	1.83	23.2	22.4	16.47*	<0.05
miRNA-3095-3p	AAAAGCTCTCTCTCCAGT	2.72	45.5	69.9	34.20*	<0.05
miRNA-181d-5p	ACCCACCGACAACAATG	0.88	2.22	1.68	2.06*	<0.05
miRNA-3091-5p	GCGGGCCCAACC	1.02	43.8	12.8	23.51*	<0.05

*The data of the SAMR1-10wk group are used as the reference value (1.0)*.

*miRNA, microRNA; SAMP, senescence-accelerated mouse prone; SAMR, senescence-accelerated mouse resistant; wk, weeks old*.

* *miRNAs whose expressions had a significant difference in the Tukey-Kramer analysis between SAMR1-10wk +SAMR1-50wk and SAMP1-10wk+SAMP1-50wk*.

**Table 1B:** miRNAs whose expressions were significantly higher (by >2.0 times) in SAMP1 mouse kidneys compared to SAMR1 mouse kidneys by microarray (SAMR1-10wk + SAMP1-10wk vs. SAMR1-50wk + SAMP1-50wk).

**MicroRNA**	**Sequence**	**SAMR1-50wk**	**SAMP1-10wk**	**SAMP1-50wk**	**Fold change (50wk mice/10wk mice)**	***p*** **(ANOVA)**
miRNA-802-5p	AAGGATGAATCTTTGTTACTGA	17.0	16.4	18.0	4.31*	<0.05

*The data of the SAMR1-10wk group are used as the reference value (1.0)*.

*miRNA, microRNA; SAMP, senescence-accelerated mouse prone; SAMR, senescence-accelerated mouse resistant; wk, weeks old*.

* *miRNAs whose expressions had a significant difference in the Tukey-Kramer analysis between SAMR1-10wk +SAMR1-50wk and SAMP1-10wk+SAMP1-50wk*.

**Table 1C:** miRNAs whose expressions were significantly higher (by >2.0 times) in the comparison of four groups of kidneys by microarray (SAMR1-10wk vs. SAMR1-50wk vs. SAMP1-10wk vs. SAMP1-50wk).

**MicroRNA**	**Sequences**	**SAMR1-50wk**	**SAMP1-10wk**	**SAMP1-50wk**	**Fold change**	***p***
let-7d-3p	AGAAAGGCAGCAGGTCGT	6.13	5.80	4.69	•5.80* (SAMP1-10wk/ SAMR1-10wk) •6.13* (SAMR1-50wk/SAMR1-10wk) •4.69* (SAMP1-50wk/SAMR1-10wk)	<0.05
miRNA-142a-3p	TCCATAAAGTAGGAAACACTACA	1.03	1.22	3.10	•3.01* (SAMP1-50wk/SAMR1-50wk) •2.55* (SAMP1-50wk/SAMP1-10wk) •3.10* (SAMP1-50wk/SAMR1-10wk)	<0.05
miRNA-142a-5p	AGTAGTGCTTTCTACTTTA	1.04	1.26	3.44	•3.31* (SAMP1-50wk/SAMR1-50wk) •2.72* (SAMP1-50wk/SAMP1-10wk) •3.44* (SAMP1-50wk/SAMR1-10wk)	<0.05
miRNA-155-5p	ACCCCTATCACAATTAGC	0.48	1.08	1.34	•2.77* (SAMP1-50wk/SAMR1-50wk) •−2.07* (SAMR1-50wk/SAMR1-10wk) •−2.23* (SAMR1-50wk/SAMP1-10wk)	<0.05
miRNA-144-3p	AGTACATCATCTATACTGTA	0.01	0.07	1.78	•−13.8* (SAMP1-10wk/SAMR1-10wk) •301.0* (SAMP1-50wk/SAMR1-50wk) •24.62* (SAMP1-50wk/SAMP1-10wk) •−168.8* (SAMR1-50wk/SAMR1-10wk) •−12.2* (SAMR1-50wk/SAMP1-10wk)	<0.05
miRNA-451a	AACTCAGTAATGGTAACGGTTT	0.10	0.37	1.70	•−2.68* (SAMP1-10wk/SAMR1-10wk) •−9.79* (SAMR1-50wk/SAMR1-10wk) •16.64* (SAMP1-50wk/SAMR1-50wk) •4.55* (SAMP1-50wk/SAMP1-10wk) •−3.66* (SAMR1-50wk/SAMP1-10wk)	<0.05

*The data of the SAMR1-10wk group are used as the reference value (1.0)*.

*miRNA, microRNA; SAMP, senescence-accelerated mouse prone; SAMR, senescence-accelerated mouse resistant; wk, weeks old*.

* *miRNAs whose expressions had a significant difference in the Tukey-Kramer analysis between SAMR1-10wk +SAMR1-50wk and SAMP1-10wk+SAMP1-50wk*.

**Figure 3:**
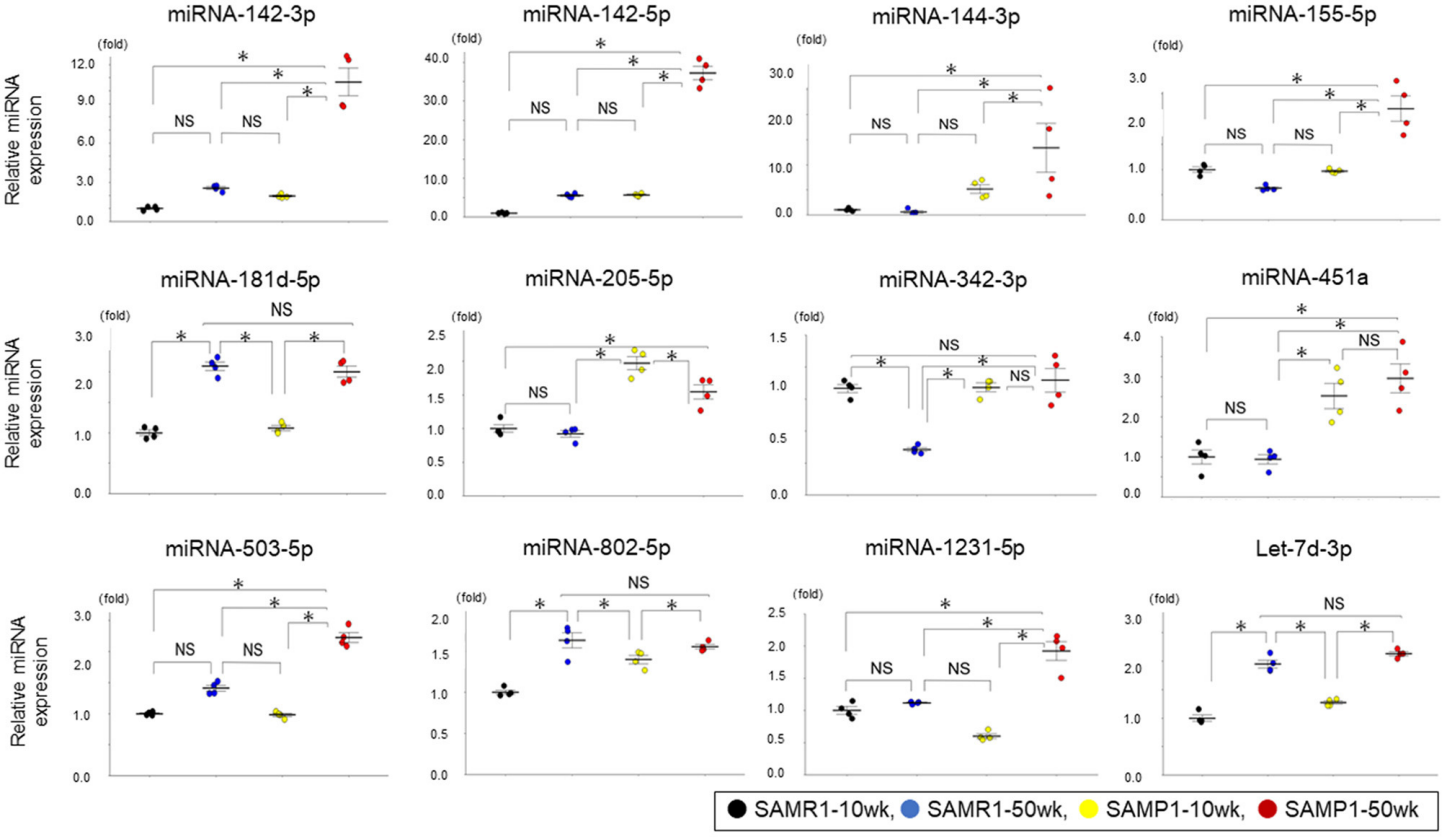
Expressions of miRNAs analyzed by qRT-PCR in the kidney of mice. MiRNA expressions of miRNA-142a-3p,−142a-5p,−144-3p,−155-5p,−181d-5p, −205-5p,−342-3p,−451a,−503-5p,−802-5p,−1231-5p, and let-7d-3p in SAMP1-50wk mice were upregulated than those of the SAMR1-10wk mice, SAMR1-50wk mice, and SAMP1-10wk mice (*n* = 4 each group). Values represent mean ± SE (error bars). *p < 0.05, ANOVA, Tukey's test.

We identified a total of 16 miRNAs whose expressions in one group were increased by >twofold or decreased by >50% compared to other groups: miRNA-142a-3p,−142a-5p,−144-3p,−155-5p,−181d-5p,−205-5p,−342-3p,−451a,−467a-3p,−503-5p,−802-5p,−1231-5p,−3091-5p,−3095-3p,−6981-5p, and let-7d-3p. No miRNAs in the SAMP1-50wk group were expressed at a level <50% of that in the SAMR1-10wk, SAMP1-10wk, and SAMR1-50wk groups (Tables 1A–C). We confirmed the expression level of 12 miRNAs that are present in humans (miRNA-142a-3p,−142a-5p,−144-3p,−155-5p,−181d-5p,−205-5p,−342-3p,−451a,−503-5p,−802-5p,−1231-5p, and let-7d-3p) in the four mouse groups by conducting a qRT-PCR. This analysis demonstrated that the expression levels of six miRNAs (miRNA-142a-3p,−142a-5p,−144-3p,−155-5p,−503-5p, and−1231-5p) were significantly upregulated in the SAMP1-50wk group in comparison to the controls, i.e., SAMR1-10wk, SAMR1-50wk, and SAMP1-10wk groups (Figure 3). No miRNA in the SAMP1-50wk group was downregulated compared to the controls (Figure 3).

### miRNA-503-5p Expression in the Kidney of Other Model Mice

**Figure 4:**
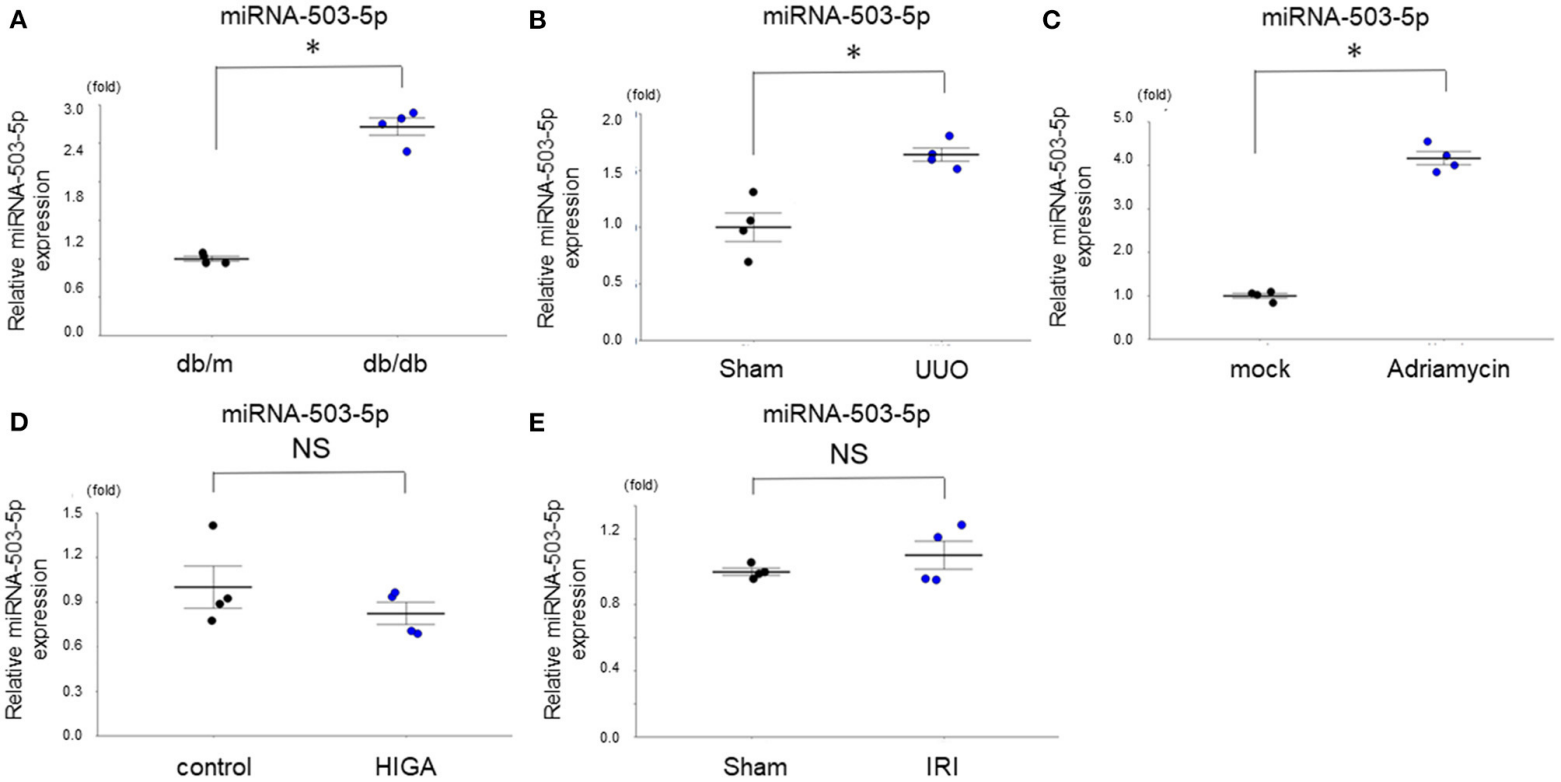
Expression levels of miRNA-503-5p in the kidney of various mouse models of kidney disease (*n* = 4 per group). **(A)** Expression levels of miRNA-503-5p in the kidney of mouse models of diabetic kidney disease (*n* = 4 per group). **(B)** Expression levels of miRNA-503-5p in the kidney of mouse models of renal fibrosis caused by UUO (*n* = 4 per group). **(C)** Expression levels of miRNA-503-5p in the kidney of mouse models of glomerulosclerosis generated by Adriamycin intravenously injected (*n* = 4 per group). **(D)** Expression levels of miRNA-503-5p in the kidney of various models of acute kidney injury (ischemia reperfusion injury) (*n* = 4 per group). (E) Expression levels of miRNA-503-5p in the kidney of mouse models of immunoglobulin A nephropathy (*n* = 4 per group). *p < 0.05, Student's *t*-test. HIGA: high immunoglobulin A, IRI: ischemia-reperfusion injury, NS: not significant, UUO: unilateral ureteral obstruction.

The expression level of one miRNA (miRNA-503-5p) that we observed was differentially expressed in kidneys of the present study's age-dependent renal impairment mice was also evaluated in kidneys of other model mice of kidney diseases by qRT-PCR: a model of diabetic kidney disease (DKD) (db/db mice) (15), a model of renal fibrosis caused by UUO (6), a model of acute kidney injury (ischemia-reperfusion injury; IRI) (16), a model of focal segmental glomerulosclerosis (FSGS) generated by adriamycin intravenous injection (17), and a model of immunoglobulin A nephropathy (HIGA/NscSlc) (18). The expression level of miRNA-503-5p differed between the DKD, UUO, and FSGS mice and the respective groups of control mice (Figures 4A–C), while it did not differ between the IRI and HIGA mice and the respective control groups (Figures 4D,E). These results indicate the various expression of miRNA-503-5p in the kidney may not be specific to age-dependent renal impairment; rather, it may be changed in lesions that cause renal fibrosis or glomerulosclerosis.

### Association Between Serum miRNA Expression Evaluated by qRT-PCR

The baseline characteristics of the 13 patients with age-dependent renal impairment (eGFR <60 mL/min/1.73 m^2^) are shown in Tables 2A,B. The patients' renal function was 40.5 ± 12.0 mL/min/1.73 m^2^ (cystatin C). We investigated the six miRNAs (miRNA-142-3p,−142-5p,−144-3p,−155-5p,−503-5p, and−1231-5p) whose expression was identified as upregulated in the SAMP1-50wk mice.

**Table 2A:** The baseline characteristics of the age-dependent renal impairment patients.

**Baseline characteristic**	**Age-dependent renal impairment (*n* = 13)**	**Healthy elderly (*n* = 13)**
Age, years	69.4 ± 4.07	69.6 ± 4.48
Male/female	12/1	13/0
eGFR cys, mL/min/1.73m^2^	40.5 ± 12.0	80.9 ± 13.2

*eGFR, estimated glomerular filtration rate*.

**Table 2B:** Characteristics of the propensity score matched pairs.

**Age-dependent renal impairment group**					**Healthy elderly group**				
**Pt**.	**Age, years**	**Sex**	**eGFR**	**Propensity score**	**Pt**.	**Age, years**	**Sex**	**eGFR**	**Propensity score**
5	70	M	37.7	0.52	105	69	M	81.8	0.43
10	68	M	58.3	0.35	87	67	M	63.5	0.27
16	65	M	47.7	0.16	111	65	M	72.1	0.16
26	76	M	23.8	0.90	93	76	M	87.1	0.90
28	66	M	36.4	0.21	104	66	M	79.5	0.27
30	76	F	14.9	0.97	90	80	M	89.2	0.97
42	73	M	35.9	0.75	95	73	M	84.7	0.75
53	72	M	45.4	0.68	102	71	M	94.1	0.60
61	68	M	48.2	0.35	100	68	M	64.5	0.35
64	66	M	55.6	0.21	94	66	M	112.1	0.21
66	65	M	46.5	0.16	103	66	M	69.5	0.21
67	65	M	36.3	0.16	99	66	M	74.3	0.21
71	72	M	40.3	0.68	91	71	M	79.1	0.60

*eGFR, estimated glomerular filtration rate*.

**Figure 5:**
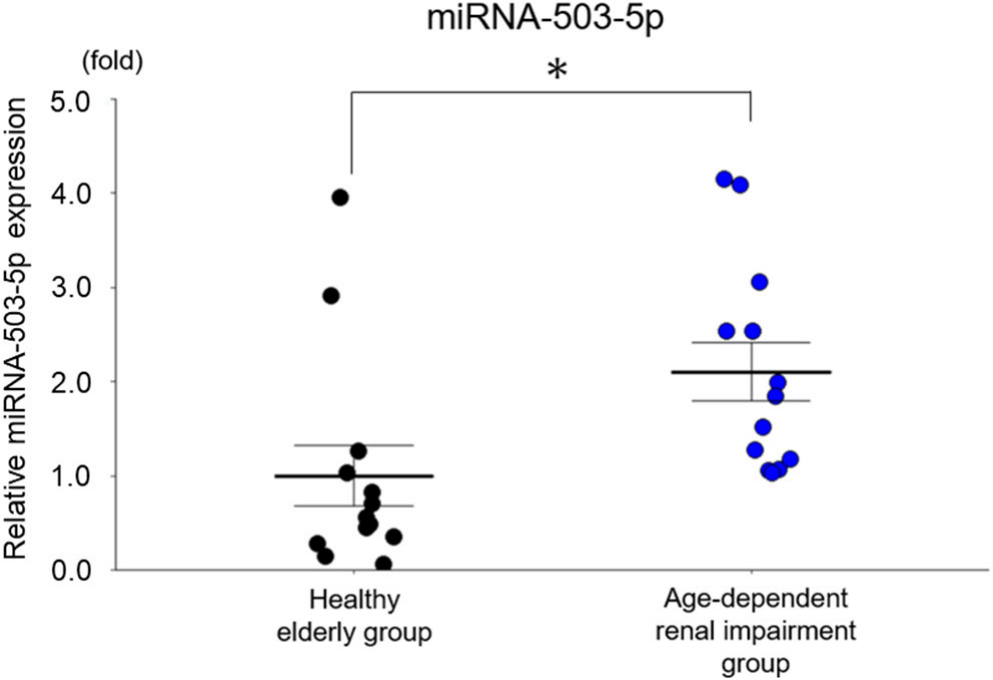
The expression of miRNA-503-5p in the serum of healthy subjects and older patients with age-dependent renal impairment. miRNA-503-5p expression in each group (*n* = 13) Values represent mean ± SE (error bars). Student's test. *p < 0.05.

We first evaluated the expressions of these six miRNAs between the age-dependent renal impairment patients and the age-matched subjects with normal renal function. The expressions of serum miRNA-142a-3p, miRNA-142a-5p, miRNA-144-3p, miRNA-155-5p, and miRNA-1231-5p showed no significant between-group difference. The expression of serum miRNA-503-5p was significantly upregulated in the patients with age-dependent renal impairment compared to that of the age-matched subjects with normal renal function (*p* = 0.01, Figure 5).

### Relative miRNA-503-5p Expression in SAMP1 Mice

**Figure 6:**
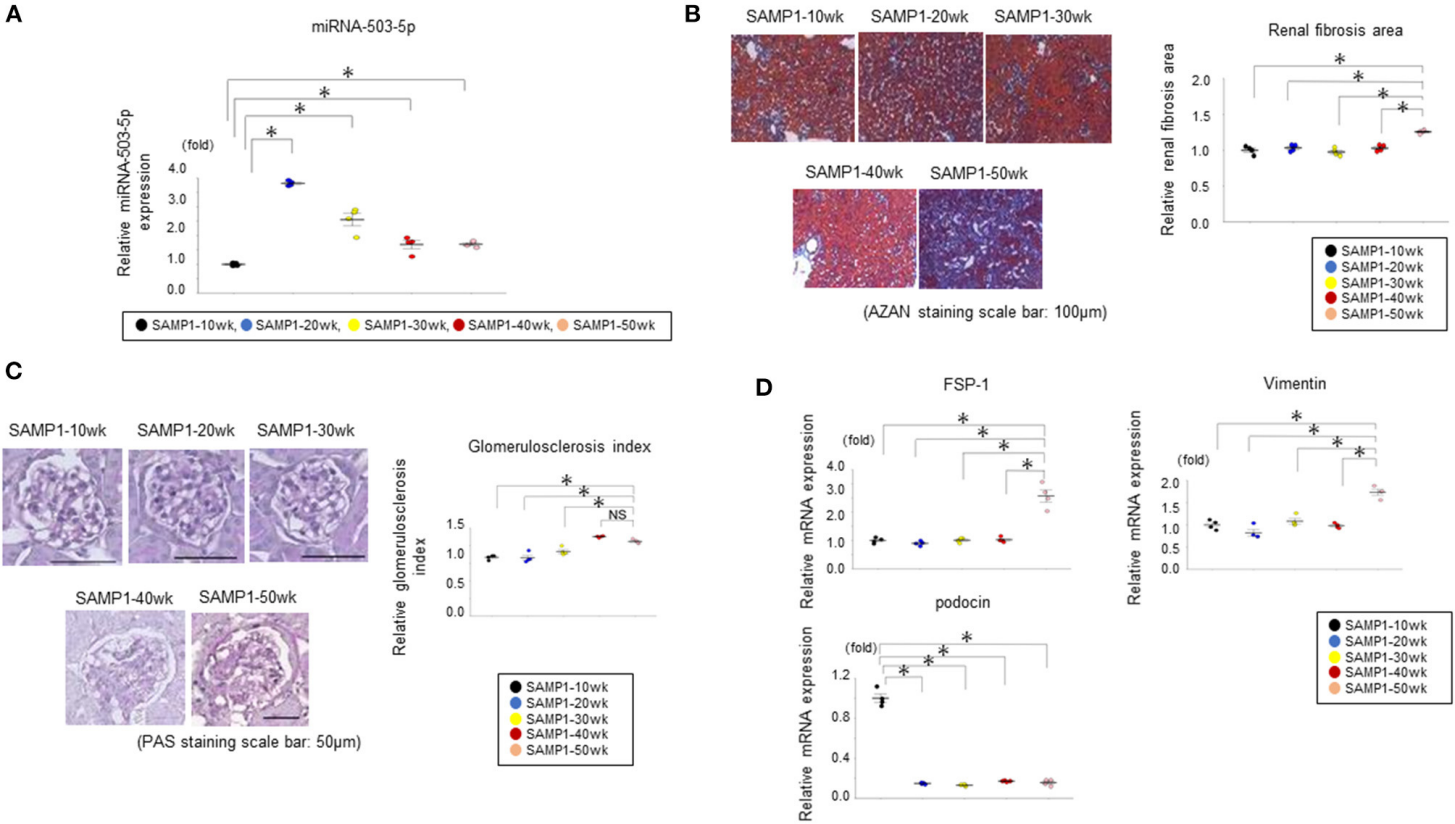
**(A)** Results of the qRT-PCR analysis of miRNA-503-5p in kidneys of SAMP1 mice of different ages. miRNA-503-5p expression in each group (*n* = 4). Values represent mean ± SE (error bars). *p < 0.05, ANOVA, Tukey's test. **(B)** Changes of interstitial nephropathy in the SAMP1-10wk mice, SAMP1-20wk mice, SAMP1-30wk mice, SAMP1-40wk mice, and SAMP1-50wk mice (*n* = 4 each). Azan staining paraffin sections. Magnification: ×200. Scale bar: 100 μm. Marked renal fibrosis was observed in SAMP1-50wk mice. Values represent mean ± SE (error bars). *p < 0.05, ANOVA, Tukey's test. **(C)** Changes in the glomerulosclerosis in SAMP1-10wk mice, SAMP1-20wk mice, SAMP1-30wk mice, SAMP1-40wk mice, and SAMP1-50wk mice. Periodic acid-Schiff-stained paraffin sections. Magnification: ×200. Scale bar: 50 μm. Marked glomerulosclerosis was observed in the SAMP1-40wk mice and SAMP1-50wk mice. Values represent mean ± SE (error bars). *p < 0.05, ANOVA, Tukey's test. **(D)** Changes in a fibrosis marker and glomerulosclerosis marker in SAMP1-10wk mice, SAMP1-20wk mice, SAMP1-30wk mice, SAMP1-40wk mice, and SAMP1-50wk mice. Results of the qRT-PCR analysis of the expression of fibroblast specific protein-1 (FSP-1), vimentin, and podocin in each group (*n* = 4). Values represent mean ± SE (error bars). *p < 0.05, ANOVA, Tukey's test.

The level of miRNA-503-5p expression was upregulated in the SAMP1-20wk mouse group (Figure 6A).

### Evaluation of Renal Fibrosis and Glomerulosclerosis in the SAMP1 Mice

Histologically, SAMP1-50wk mice demonstrated typical features of fibrosis compared to the SAMP1-10wk mice, SAMP1-20wk mice, SAMP1-30wk mice, and SAMP1-40wk mice (Figure 6B). In addition, SAMP1-40wk mice and SAMP1-50wk mice demonstrated typical features of glomerulosclerosis compared to the SAMP1-10wk mice, SAMP1-20wk mice, and SAMP1-30wk mice (Figure 6C). The results of the qRT-PCR analysis confirmed further increases in the renal fibrosis markers vimentin and FSP-1 in the SAMP1-50wk mice, and that the expression of the glomerulosclerosis marker podocin was decreased in the SAMP1-20wk mice, SAMP1-30wk mice, 40-wk mice, and SAMP1-50wk mice (Figure 6D).

### Transfection of miRNA in the Age-Dependent Renal Impairment Kidneys by miRNA-PEI-NPs

**Figure 7:**
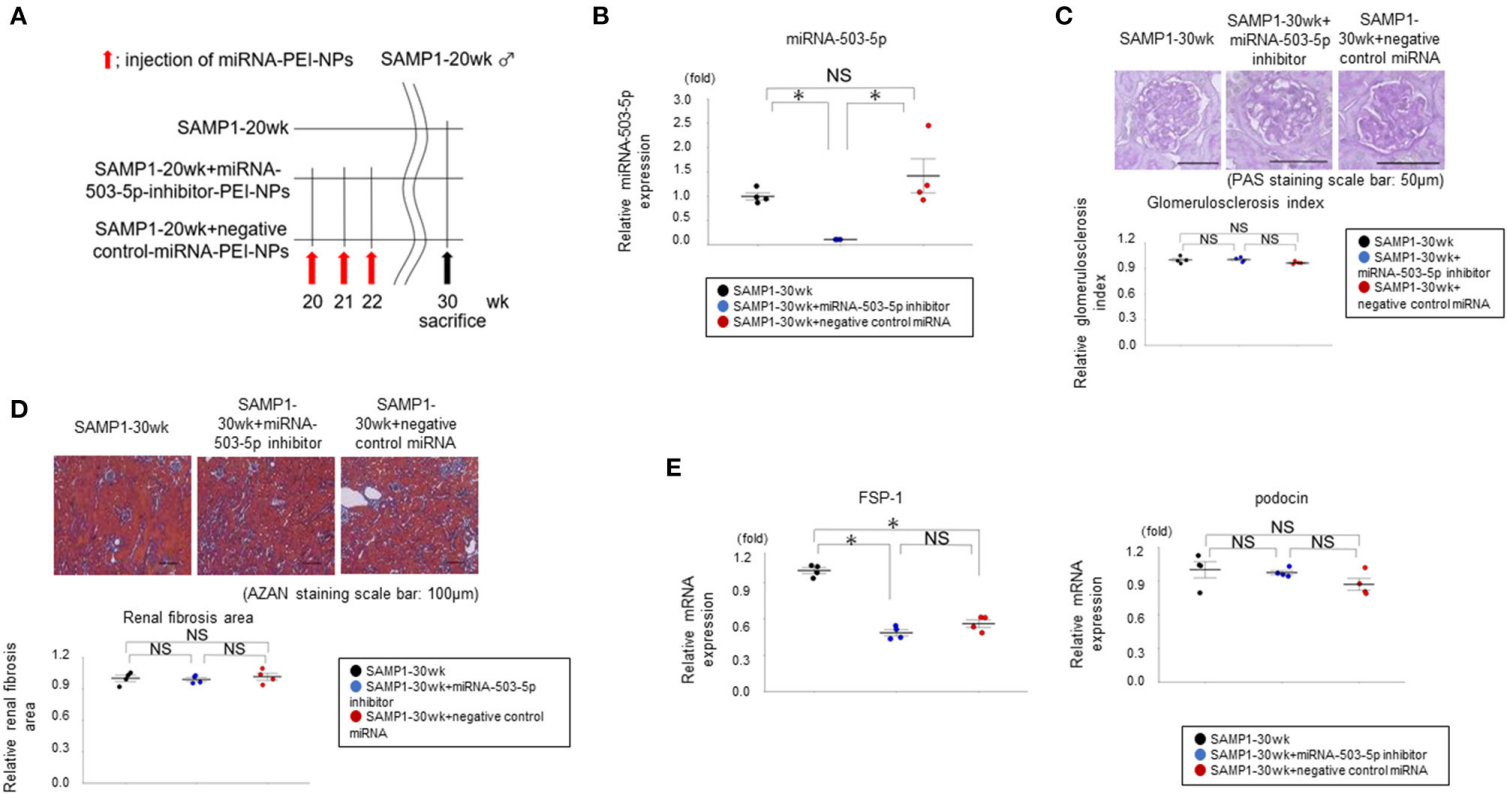
**(A)** Protocol for administration of miRNA-503-5p-inhibitor-PEI-NPs to SAMP1-20wk mice. **(B)** Results of the qRT-PCR analysis of the inhibition of miRNA-503-5p in SAMP1 mice kidneys following an injection of miRNA-503-5p-inhibitor-PEI-NPs. miRNA-503-5p expression in each group (*n* = 4). Values represent mean ± SE (error bars). *p < 0.05, ANOVA, Tukey's test. **(C)** Changes in glomerulosclerosis in the SAMP1-30wk mice, SAMP1-30wk+miRNA-503-5p-inhibitor-PEI-NP mice, and SAMP1-30wk+negative control miRNA-PEI-NP mice (*n* = 4 each). Periodic acid-Schiff-stained paraffin section. Magnification: ×200. Scale bar: 50 μm. No improvement of glomerulosclerosis was observed in the SAMP1-30wk+miRNA-503-5p-inhibitor-PEI-NP mice. Values represent mean ± SE (error bars). *p < 0.05, ANOVA, Tukey's test. **(D)** Changes in the fibrosis of SAMP1-30wk mice, SAMP1-30wk+miRNA-503-5p-inhibitor-PEI-NP mice, and SAMP1-30wk+negative control miRNA-PEI-NP mice (*n* = 4 each). Azan staining sections. Magnification: ×200. Scale bar: 100 μm. No improvement of fibrosis was observed in the SAMP1-30wk+miRNA-503-5p-inhibitor-PEI-NP mice. Values represent mean ± SE (error bars). *p < 0.05, ANOVA, Tukey's test. **(E)** Changes in the values of a fibrosis marker and glomerulosclerosis marker in SAMP1-30wk mice, SAMP1-30wk+miRNA-503-5p-inhibitor-PEI-NP mice, and SAMP1-30wk+negative control miRNA-PEI-NP mice. The results of the qRT-PCR analysis of expression of podocin and FSP-1 in each group (*n* = 4 each). Values represent mean ± SE (error bars). *p < 0.05, ANOVA, Tukey's test.

The miRNA-503-5p-inhibitor-PEI-NPs administration protocol for SAMP1-20wk mice is shown in Figure 7A. The administration of miRNA-503-5p-inhibitor-PEI-NPs, but not control-miRNA-PEI-NPs, demonstrated a significant decrease in the expression of miRNA-503-5p in the age-dependent renal impairment kidneys (Figure 7B).

### Effects of miRNA-PEI-NPs on Fibrosis Markers and Glomerulosclerosis Markers in Age-Dependent Renal Impairment Kidneys

Histologically, compared to the SAMP1-30wk mice and the SAMP1-30wk+control-miRNA-PEI-NP mice, the SAMP1-30wk+miRNA-503-5p-inhibitor-PEI-NP mice showed no improvement of glomerulosclerosis (Figure 7C). In addition, compared to the SAMP1-30wk mice and SAMP1-30wk+control-miRNA-PEI-NP mice, the SAMP1-30wk+miRNA-503-5p-inhibitor-PEI-NP mice showed no improvement of fibrosis (Figure 7D). The results of the qRT-PCR analysis demonstrated that the expressions of fibrosis markers and glomerulosclerosis markers were not significantly different between the SAMP1-30wk+miRNA-503-5p-inhibitor-PEI-NP group and the SAMP1-30wk+control-miRNA-PEI-NP group (Figure 7E).

### Transfection of miRNA in the Obstructed Kidneys by miRNA-PEI-NPs

**Figure 8:**
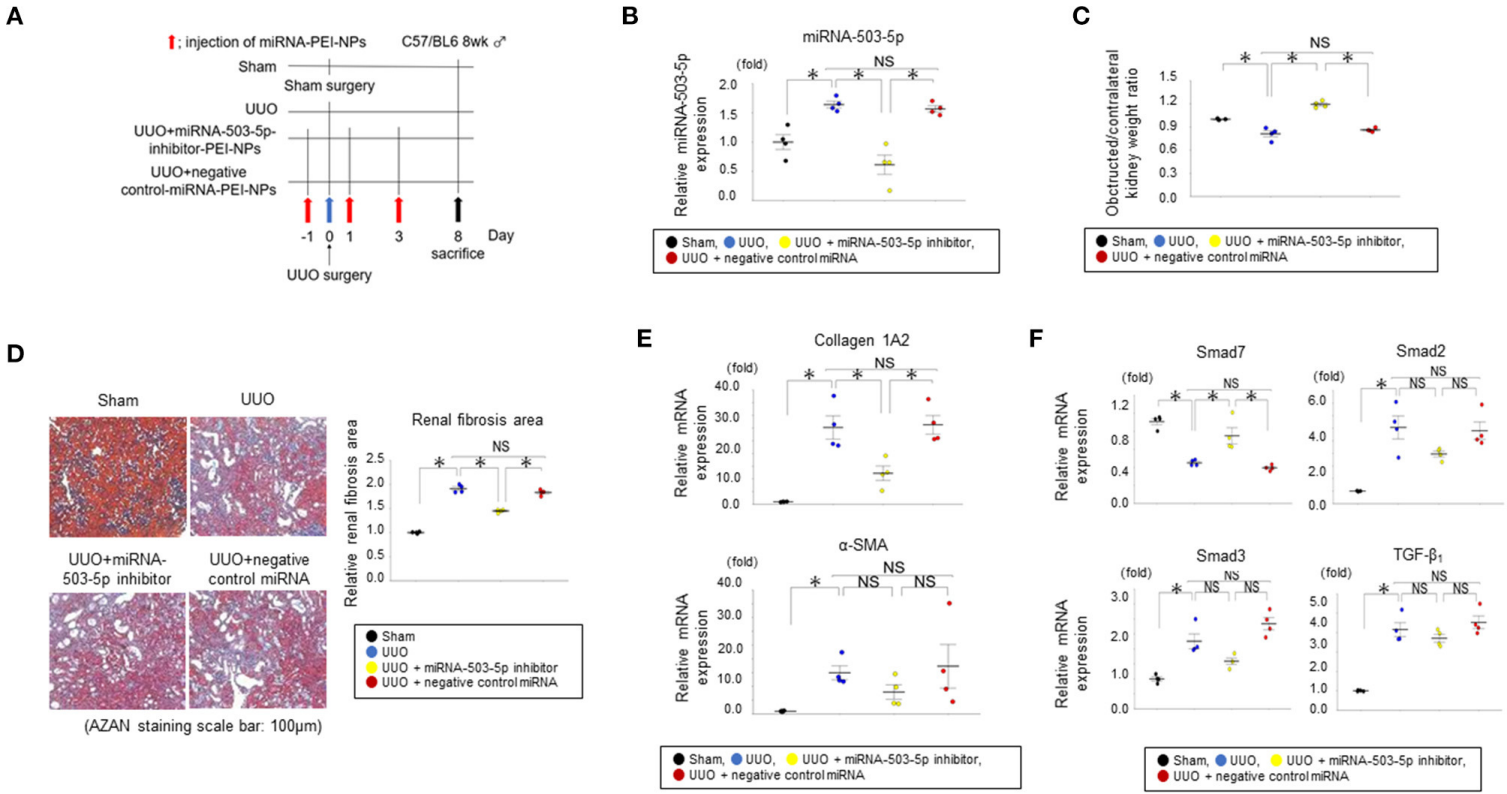
**(A)** Protocol for administration of miRNA-503-5p-inhibitor-PEI-NPs to UUO mice. UUO: unilateral ureteral obstruction. **(B)** Results of the qRT-PCR analysis of the inhibition of miRNA-503-5p in obstructed kidneys following injections of miRNA-503-5p-inhibitor-PEI-NPs. miRNA-503-5p expression in each group (*n* = 4 each). Values represent mean ± SE (error bars). *p < 0.05, ANOVA, Tukey's test. UUO: unilateral ureteral obstruction. **(C)** Effects of miRNA-503-5p-inhibitor-PEI-NPs on kidney weight. Ratios of obstructed kidney weight to contralateral kidney weight in each group (n=4 each). Values represent mean ± SE (error bars). *p < 0.05, ANOVA, Tukey's test. **(D)** Effects of miRNA-503-5p-inhibitor-PEI-NPs on fibrous changes in obstructed kidneys (n=4 each). Azan staining paraffin sections. Magnification: ×200. Scale bar: 100 μm. Values represent mean ± SE (error bars). *p < 0.05, ANOVA, Tukey's test. **(E)** Effects of miRNA-503-5p-inhibitor-PEI-NPs on fibrotic markers. Results of the qRT-PCR analysis of expression of collagen 1A2 and alpha-smooth muscle actin (α-SMA) in each group (*n* = 4 each). Values represent mean ± SE (error bars). *p < 0.05, ANOVA, Tukey's test. **(F)** Effects of miRNA-503-5p-inhibitor-PEI-NPs on the expressions of Smad7, Smad2, Smad3, and TGF-β_1_ in obstructed kidneys. Results of the qRT-PCR analysis of Smad7, Smad2, Smad3, and TGF-β_1_ expression in each group (*n* = 4 each). Values represent mean ± SE (error bars). *p < 0.05, ANOVA, Tukey's test. Smad, small mothers against decapentaplegic; TGF, transforming growth factor.

The miRNA-503-5p-inhibitor-PEI-NPs administration protocol for UUO mice is shown in Figure 8A. The administration of miRNA-503-5p-inhibitor-PEI-NPs, but not control-miRNA-PEI-NPs, demonstrated a significant decrease in the expression of miRNA-503-5p in the obstructed kidneys (Figure 8B).

### Effects of miRNA-PEI-NPs on the Obstructed Kidney Weight

The ratio of the weight of obstructed kidneys in each group to that of the contralateral non-obstructed kidneys are depicted in Figure 8C. The ratio was considerably downregulated in the UUO group compared to sham-surgery group. This downregulation was suppressed by the administration of miRNA-503-5p-inhibitor-PEI-NPs, but not by administration of control-miRNA-PEI-NPs.

### Inhibition of Renal Fibrosis by miRNA-PEI-NPs

The positive blue-stained areas demonstrating collagen fibers with azan staining were increased considerably in the obstructed kidneys in the UUO + non-injection group compared to the sham-surgery group (Figure 8D). These fibrous areas were considerably improved by the administration of miRNA-503-5p-inhibitor-PEI-NPs, but not by control-miRNA-PEI-NPs. Fibrous changes in each group were observed by azan staining and quantification, with an average quantified fibrotic areas per field in the UUO + non-injection group at 1.81 ± 0.03, the UUO + miRNA-503-5p-inhibitor-PEI-NP group at 1.50 ± 0.03, and the UUO + control-miRNA-PEI-NP group at 1.79 ± 0.05 compared with the sham-operation group (Figure 8D). Also, the increase in fibrotic areas in obstructed kidneys was significantly suppressed by miRNA-503-5p-inhibitor-PEI-NPs, but not by control-miRNA-PEI-NPs (Figure 8D).

### Effects of miRNA-PEI-NPs on Fibrosis Markers and pro-Fibrotic Signals in Obstructed Kidneys

The qRT-PCR analysis demonstrated that the expression of collagen 1A2 mRNA was upregulated in the obstructed kidneys in relation to the sham-surgery kidneys (Figure 8E). These increases were considerably suppressed by miRNA-503-5p-inhibitor-PEI-NPs, but not by control-miRNA-PEI-NPs. The expression level of α-SMA mRNA demonstrated a similar trend, but these changes were not statistically significant (Figure 8E). Smad7 is reported to be a target gene of miRNA-503-5p (19). Our qRT-PCR analysis demonstrated that the expression level of Smad7 mRNA was lowered in the obstructed kidneys compared to the sham-surgery kidneys (Figure 8F). These decreases were also significantly inhibited by miRNA-503-5p-inhibitor-PEI-NPs, but not by control-miRNA-PEI-NPs.

We also evaluated changes in the expressions of Smad2, Smad3, and TGF-β_1_, which are considered valuable signaling molecules in the progression of renal fibrosis. The expressions of Smad2, Smad3, and TGF-β_1_ were upregulated in the obstructed kidneys compared to the sham-surgery kidneys (Figure 8F). These increases were not significantly lowered by miRNA-503-5p-inhibitor-PEI-NPs (Figure 8F).

### Transfection of miRNA in the Adriamycin-Injected Kidneys by miRNA-PEI-NPs

**Figure 9:**
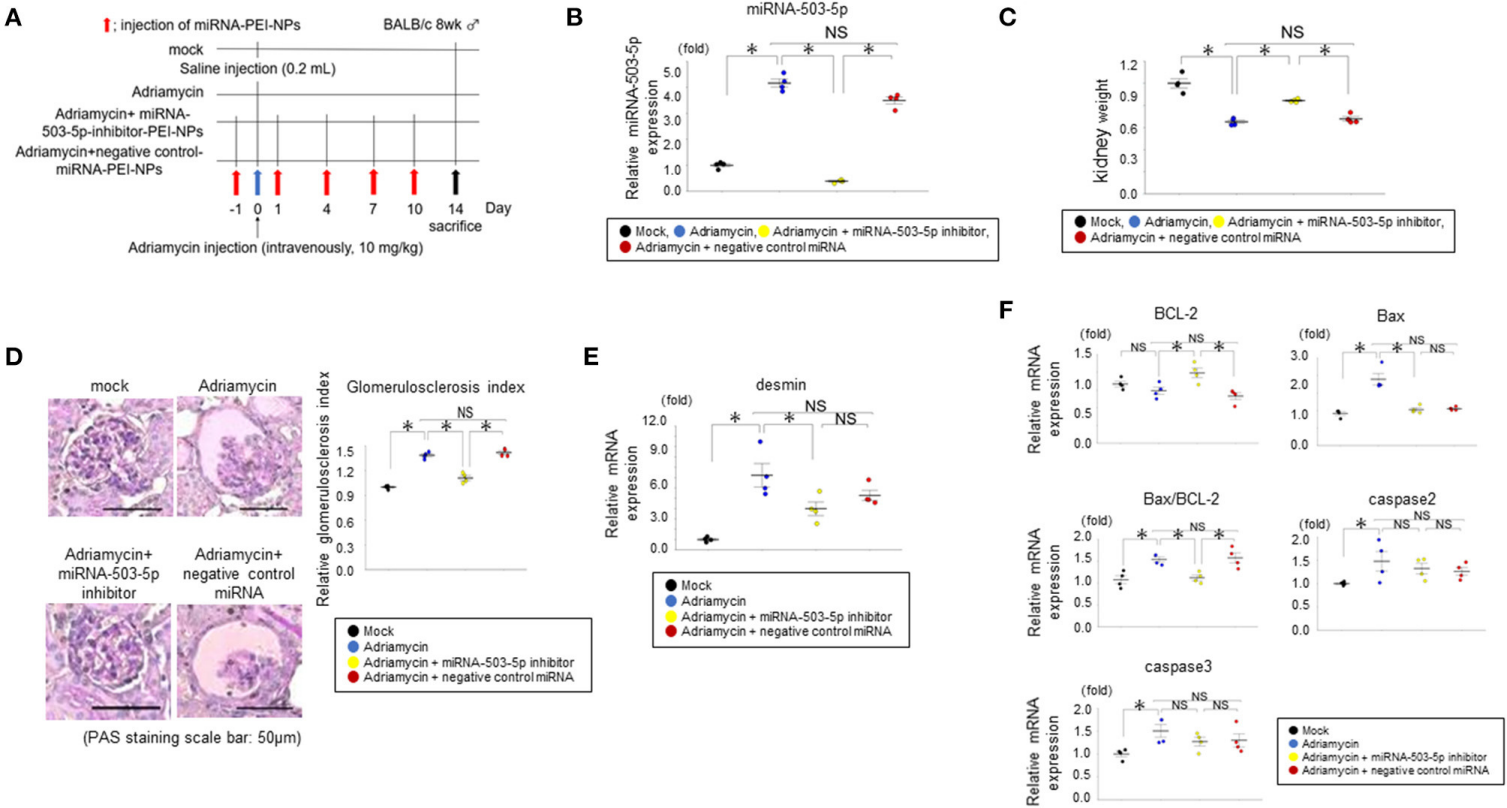
**(A)** Protocol for administration of miRNA-503-5p-inhibitor-PEI-NPs to adriamycin mice. **(B)** Results of qRT-PCR analysis of the inhibition of miRNA-503-5p in adriamycin kidneys following an injection of miRNA-503-5p-inhibitor-PEI-NPs. miRNA-503-5p expression in each group (*n* = 4 each). Values represent mean ± SE (error bars). *p < 0.05, ANOVA, Tukey's test. **(C)** Effects of miRNA-503-5p-inhibitor-PEI-NPs on kidney weight. Ratios of adriamycin kidney weight to contralateral kidney weight in each group (*n* = 4 each). Values represent mean ± SE (error bars). *p < 0.05, ANOVA, Tukey's test. **(D)** Effects of miRNA-503-5p-inhibitor-PEI-NPs on glomerulosclerosis changes in adriamycin kidneys (*n* = 4 each). Periodic-acid Schiff staining paraffin sections. Magnification: ×200. Scale bar: 50 μm. Values represent mean ± SE (error bars). *p < 0.05, ANOVA, Tukey's test. **(E)** Changes in a glomerulosclerosis marker in adriamycin-injected mice. Results of the qRT-PCR analysis of expression of desmin in each group (*n* = 4 each). Values represent mean ± SE (error bars). *p< 0.05, ANOVA, Tukey's test. **(F)** Effects of miRNA-503-5p-inhibitor-PEI-NPs on the expression of B-cell lymphoma-2 (BCL-2), caspase 2, and caspase 3 in adriamycin kidneys. Results of the qRT-PCR analysis of BCL-2, caspase 2, and caspase 3 expression in each group (*n* = 4 each). Values represent mean ± SE (error bars). *p < 0.05, ANOVA, Tukey's test.

The miRNA-503-5p-inhibitor-PEI-NPs administration protocol for adriamycin mice is shown in Figure 9A. The administration of miRNA-503-5p-inhibitor-PEI-NPs, but not control-miRNA-PEI-NPs, demonstrated a significant decrease in the expression of miRNA-503-5p in the kidneys of the adriamycin-injected mice (Figure 9B).

### Effects of miRNA-PEI-NPs on the Adriamycin-Injected Kidney Weight

The ratio of the weight of the adriamycin-injected kidneys to that of uninjected kidneys in each group are shown in Figure 9C. The ratio was significantly lowered in the adriamycin-injected mice compared to the mock group. This difference was suppressed by the administration of miRNA-503-5p-inhibitor-PEI-NPs, but not of control-miRNA-PEI-NPs.

### Effects of miRNA-503-5p-Inhibitor-PEI-NPs on Glomerulosclerosis and Apoptosis Mediators in Adriamycin Kidneys

There was a significant increase in the glomerulosclerosis index in the adriamycin nephropathy of the adriamycin + non-injection group compared to the mock group (Figure 9D). This pathological change was significantly improved by the administration of miRNA-503-5p-inhibitor-PEI-NPs, but not by control-miRNA-PEI-NPs. Pathological changes in each group were confirmed by PAS staining, with the mean glomerulosclerosis index values as follows: the adriamycin + non-injection group, 1.39 ± 0.02; adriamycin + miRNA-503-5p-inhibitor-PEI-NP group, 1.11 ± 0.02; and adriamycin + control-miRNA-PEI-NP group, 1.41 ± 0.02 compared with the mock group (Figure 9D), again showing that the pathological change in adriamycin nephropathy was significantly inhibited by miRNA-503-5p-inhibitor-PEI-NPs, but not by control-miRNA-PEI-NPs (Figure 9D). The qRT-PCR analysis results demonstrated that the expression of desmin mRNA was not lowered in the adriamycin + non-injection group compared to the mock kidneys (Figure 9E).

BCL-2 has been reported to be a target of miRNA-503-5p (20). Our qRT-PCR analysis revealed that the expression of BCL-2 was upregulated in the adriamycin nephropathy kidneys than in the mock kidneys, and this increase was significantly suppressed by the miRNA-503-5p-inhibitor-PEI-NPs, but not by the control-miRNA-PEI-NPs (Figure 9F).

We also evaluated changes in the expressions of caspase 2 and caspase 3, which are considered pivotal signaling molecules in the progression of apoptosis (21). The expression levels of caspase 2 and caspase 3 were also upregulated in the adriamycin kidneys than in the mock kidneys (Figure 9F), but these increases were not significantly suppressed by miRNA-503-5p-inhibitor-PEI-NPs or control-miRNA-PEI-NPs (Figure 9F).

## Discussion

In this study, the serum levels of miRNA-503-5p were decreased in patients with age-dependent renal impairment. However, the inhibition of miRNA-503-5p had no effect on age-dependent renal impairment, although the inhibition of miRNA-503-5p had a therapeutic effect on renal fibrosis by inhibiting pro-fibrotic (Smad7–TGF-β_1_) signaling pathways and glomerulosclerosis by suppressing (Bax/BCL-2 axis) signaling pathways in an *in vivo* animal model. These results indicate that miRNA-503-5p is related to age-dependent renal impairment. Additionally. miRNA-503-5p may play a pivotal part in the progression of renal fibrosis and glomerulosclerosis, and the therapeutic effect of miRNA-503-5p-inhibitor-PEI-NPs on age-dependent renal impairment merits further investigation.

The previous study reported that the inhibition of miRNA-503-5p in cultured tubular interstitial fibroblasts in mice suppressed fibrosis (22). Another study reported that an inhibition of miRNA-503-5p in cultured human kidney-2 (HK-2) cells was involved in the suppression of diabetic nephropathy through the suppression of apoptosis (23). However, the possibility that miRNA-503-5p is related to age-dependent renal impairment has not been reported to the best of our knowledge. In this study, we first used the microarray method to comprehensively screen miRNAs whose expressions differed by >twofold or by <50% in the kidneys in four types of model mice: young normal aging model mice (SAMR1-10wk), old normal aging model mice (SAMR1-50wk), young aging accelerated model mice (SAMP1-10wk), and aging accelerated model mice (SAMP1-50wk). Four of the identified miRNAs (miRNA-467a-3p,−3091-5p,−3095-3p, and−6981-5p) have not been reported to exist in humans.

Our qRT-PCR analyses identified six miRNAs (miRNA-142a-3p,−142a-5p,−144-3p,−155-5p,−503-5p,−1231-5p) as biomarker candidates for age-dependent renal impairment (Figure 3). When the expression levels in human serum were examined by qRT-PCR, only miRNA-503-5p was significantly increased in the patients with age-dependent renal impairment by ~2 fold in comparison to the healthy elderly subjects (Figure 5). These results indicate that the serum expression of miRNA-503-5p may be useful as a diagnostic biomarker for age-dependent renal impairment. However, because the number of cases is small (*n* = 13 each group), further investigations of greater numbers of cases are necessary to test our findings.

Next, although the administration of the miRNA-503-5p-inhibitor did not show a therapeutic effect on age-dependent renal impairment, i.e., in the SAMP1 mice, the administration of the miRNA-503-5p-inhibitor was observed to exert therapeutic effects on the renal fibrosis induced by UUO and the glomerulosclerosis induced by intravenous adriamycin injection. The reason why the miRNA-503-5p-inhibitor did not exhibit a therapeutic effect on age-dependent renal impairment can be explained by the studies demonstrating that the development of age-dependent renal impairment is associated with complex and diverse pathological conditions. These include renal tubulointerstitial fibrosis and glomerulosclerosis, as well as arteriosclerosis, amyloid deposition, and renal inflammaging caused by oxidative stress and proinflammatory cytokines, including interleukin-6 and tumor necrosis factor-α (3, 24–27). An miRNA-503-5p-inhibitor alone cannot regulate all of these conditions and inhibit age-dependent renal impairment.

In the present investigation, the miRNA-503-5p-inhibitor was administered to SMAP1-20wk mice until they reached the age of 30 weeks because the expression of miRNA-503-5p in the kidney increased at the same time as podocin expression decreased in the kidney of SAMP1-20wk mice. Although the miRNA-503-5p-inhibitor did not show a therapeutic effect on age-dependent renal impairment, further studies regarding the period of administration and the evaluation time are necessary and may reveal different findings.

In contrast, we observed that the administration of the miRNA-503-5p-inhibitor exerted therapeutic effects on the renal fibrosis and glomerulosclerosis (observed in age-dependent renal impairment) induced by UUO and intravenous adriamycin injection. It is thus possible that miRNA-503-5p can improve at least some of the lesions observed in age-dependent renal impairment. We plan to use differing administration periods and evaluation time points for the use of the miRNA-503-5p-inhibitor in SAMP1 mice in future studies.

We used PEI-NPs to suppress the miRNA-503-5p expression in this study; PEI-NPs are polymers that are reported to effectively deliver oligonucleotides that can modulate target gene expressions in the kidney, and they are thought to be suitable to viral vector because of their biocompatibility and long-term safety (28). In these experiments, the administration of miRNA-503-5p-inhibitor-PEI-NPs resulted in a significant inhibition of miRNA-503-5p in the SAMP1 mice, UUO mice, and mice with glomerulosclerosis induced by intravenous adriamycin injection. Additionally, inhibition of miRNA-503-5p promotes colorectal cancer by increasing the expression of vascular endothelial growth factor-A (29). In addition, inhibition of miRNA-503-5p promotes carotid artery stenosis by increasing the expression of fibroblast growth factor 2 (30). Therefore, miRNA-503-5p-inhibitor-PEI-NPs may be able to suppress age-dependent renal impairment but may also be involved in the development of colorectal cancer and carotid artery stenosis. It is therefore necessary to verify the therapeutic effects of miRNA-503-5p-inhibitor-PEI-NPs in other organs.

Our study has several limitations. The analysis of the findings might be limited by sample sizes. In addition, the changes in miRNA expressions depending on the stage of chronic kidney disease in the patient with age-dependent renal impairment were not analyzed. Lastly, the effect of miRNA-503-5p-inhibitor-PEI-NPs in other organs should also be evaluated.

## Conclusion

Our present findings demonstrate that serum levels of miRNA-503-5p were decreased in patients with age-dependent renal impairment. However, the inhibition of miRNA-503-5p had no effect on age-dependent renal impairment, although the inhibition of miRNA-503-5p had therapeutic effects on renal fibrosis and glomerulosclerosis in an *in vivo* animal model. These results indicate that miRNA-503-5p might be related to age-dependent renal impairment.

## Data Availability Statement

The datasets presented in this study can be found in online repositories. The names of the repository/repositories and accession number(s) can be found below: GEO database with accession GSE198741.

## Ethics Statement

The studies involving human participants were reviewed and approved by Jichi Medical University Ethics Committee. The patients/participants provided their written informed consent to participate in this study. The animal study was reviewed and approved by Jichi Medical University Ethics Committee.

## Author Contributions

SK, HI, AA, KH, and SO collected serum sample. YM corrected this manuscript. All authors contributed to the article and approved the submitted version.

## Funding

This work was supported by JSPS KAKENHI Grant Number 17K09708.

## Conflict of Interest

The authors declare that the research was conducted in the absence of any commercial or financial relationships that could be construed as a potential conflict of interest.

## Publisher's Note

All claims expressed in this article are solely those of the authors and do not necessarily represent those of their affiliated organizations, or those of the publisher, the editors and the reviewers. Any product that may be evaluated in this article, or claim that may be made by its manufacturer, is not guaranteed or endorsed by the publisher.

## References

[1] Hallan SI Matsushita K Sang Y Mahmoodi BK Black C Ishani A. Age and association of kidney measures with mortality and end-stage renal disease. JAMA. (2012) 308:2349–2360. 10.1001/jama.2012.16817 23111824 PMC3936348

[2] Wang G Kwan BC Lai FM Choi PC Chow KM Li PK. Intrarenal expression of miRNAs in patients with hypertensive nephrosclerosis. Am J Hypertens. (2010) 23:78–84. 10.1038/ajh.2009.208 19910931

[3] Zhou XJ Rakheja D Yu X Saxena R Vaziri ND Silva FG. The aging kidney. Kidney Int. (2008) 74:710–720. 10.1038/ki.2008.319 18614996

[4] Fabian MR Sonenberg N Filipowicz W. Regulation of mRNA translation and stability by microRNAs. Annu Rev Biochem. (2010) 79:351–379. 10.1146/annurev-biochem-060308-103103 20533884

[5] Backes C Meese E Keller A. Specific miRNA disease biomarkers in blood, serum and plasma: challenges and prospects. Mol Diagn Ther. (2016) 20:509–518. 10.1007/s40291-016-0221-4 27378479

[6] Yanai K Kaneko S Ishii H Aomatsu A Ito K Hirai K. Quantitative real-time PCR evaluation of microRNA expressions in mouse kidney with unilateral ureteral obstruction. J Vis Exp. (2020) 162. 10.3791/61383 32925880

[7] Daehn I Casalena G Zhang T Shi S Fenninger F Barasch N. Endothelial mitochondrial oxidative stress determines podocyte depletion in segmental glomerulosclerosis. J Clin Invest. (2014) 124:1608–1621. 10.1172/JCI71195 24590287 PMC3973074

[8] Kanda Y. Investigation of the freely available easy-to-use software 'EZR' for medical statistics. Bone Marrow Transplant. (2013) 48:452–8. 10.1038/bmt.2012.244 23208313 PMC3590441

[9] Morishita Y Yoshizawa H Watanabe M Ishibashi K Muto S Kusano E. siRNAs targeted to Smad4 prevent renal fibrosis *in vivo*. Sci Rep. (2014) 4:6424. 10.1038/srep06424 25236771 PMC4168270

[10] Ragni E De Luca P Perucca Orfei C Colombini A Vigano M Lugano G. Insights into inflammatory priming of adipose-derived mesenchymal stem cells: validation of extracellular vesicles-embedded miRNA reference genes as A crucial step for donor selection. Cells. (2019) 8:369. 10.3390/cells8040369 31018576 PMC6523846

[11] Liu X Zhang L Cheng K Wang X Ren G Xie P. Identification of suitable plasma-based reference genes for miRNAome analysis of major depressive disorder. J Affect Disord. (2014) 163:133–9. 10.1016/j.jad.2013.12.035 24479999

[12] Raij L Azar S Keane W. Mesangial immune injury, hypertension, and progressive glomerular damage in Dahl rats. Kidney Int. (1984) 26:137–143. 10.1038/ki.1984.147 6239058

[13] Dasgupta I Porter C Innes A Burden R. “Benign” hypertensive nephrosclerosis. QJM. (2007) 100:113–9. 10.1093/qjmed/hcl139 17244670

[14] Imai E Horio M Nitta K Yamagata K Iseki K Hara S. Estimation of glomerular filtration rate by the MDRD study equation modified for Japanese patients with chronic kidney disease. Clin Exp Nephrol. (2007) 11:41–50. 10.1007/s10157-006-0453-4 17384997

[15] Ishii H Kaneko S Yanai K Aomatsu A Hirai K Ookawara S. MicroRNA expression profiling in diabetic kidney disease. Transl Res. (2021) 237:31–52. 10.1016/j.trsl.2021.05.008 34102327

[16] Aomatsu A Kaneko S Yanai K Ishii H Ito K Hirai K. MicroRNA expression profiling in acute kidney injury. Transl Res. (2021) 4:S1931-5244(21)00283-8. 10.1016/j.trsl.2021.11.010 34871811

[17] Jiang Q He X Zou Y Ding Y Li H Chen H. Altered gut microbiome promotes proteinuria in mice induced by Adriamycin. Amb Express. (2018) 8:1–8. 10.1186/s13568-018-0558-7 29492783 PMC5833890

[18] Kaneko S Yanai K Ishii H Aomatsu A Ito K Hirai K. Detection of microRNA expression in the kidneys of immunoglobulin a nephropathic mice. JoVE. (2020) e61535. 10.3791/61535 32716396

[19] Fei Y Shan W Chen X. MiR-503-5p functions as an oncogene in oral squamous cell carcinoma by targeting Smad7. Histol Histopathol. (2020) 35:893–901. 10.14670/HH-18-220 32319077

[20] Wang T Ge G Ding Y Zhou X Huang Z Zhu W. MiR-503 regulates cisplatin resistance of human gastric cancer cell lines by targeting IGF1R and BCL2. Chin Med J. (2014) 127:2357–62. 24931256

[21] Elmore S. Apoptosis: a review of programmed cell death. Toxicol Pathol. (2007) 35:495–516. 10.1080/01926230701320337 17562483 PMC2117903

[22] Mann C Kaistha BP Kacik M Stiewe T Hoyer J. Downregulation of miR-503 in activated kidney fibroblasts disinhibits KCNN4 in an *in vitro* model of kidney fibrosis. Kidney Blood Press Res. (2019) 44:113–22. 10.1159/000498875 30808854

[23] Cao X Fan QL. LncRNA MIR503HG promotes high-glucose-induced proximal tubular cell apoptosis by targeting miR-503-5p/Bcl-2 pathway. Diabetes Metab Syndr Obes. (2020) 13:4507–17. 10.2147/DMSO.S277869 33262626 PMC7691658

[24] Tauchi H Tsuboi K Okutomi J. Age changes in the human kidney of the different races. Gerontologia. (1971) 17:87–97. 10.1159/000211811 5093734

[25] Muhlberg W Platt D. Age-dependent changes of the kidneys: pharmacological implications. Gerontology. (1999) 45:243–253. 10.1159/000022097 10460985

[26] van der Heijden RA Bijzet J Meijers WC Yakala GK Kleemann R Nguyen TQ. Obesity-induced chronic inflammation in high fat diet challenged C57BL/6J mice is associated with acceleration of age-dependent renal amyloidosis. Sci Rep. (2015) 5:16474. 10.1038/srep16474 26563579 PMC4643235

[27] Sepe V Libetta C Gregorini M Rampino T. The innate immune system in human kidney inflammaging. J Nephrol. (2021). 10.1007/s40620-021-01153-4 34826123 PMC8617550

[28] Morishita Y Imai T Yoshizawa H Watanabe M Ishibashi K Muto S. Delivery of microRNA-146a with polyethylenimine nanoparticles inhibits renal fibrosis in vivo. Int J Nanomedicine. (2015) 10:3475–88. 10.2147/IJN.S82587 25999712 PMC4435251

[29] Wei L Sun C Zhang Y Han N Sun S. miR-503-5p inhibits colon cancer tumorigenesis, angiogenesis, and lymphangiogenesis by directly downregulating VEGF-A. Gene Ther. (2022) 29:28–40. 10.1038/s41434-020-0167-3 32533103

[30] Yan Z Wang H Liang J Li Y Li X. MicroRNA-503-5p improves carotid artery stenosis by inhibiting the proliferation of vascular smooth muscle cells. Exp Ther Med. (2020) 20:85. 10.3892/etm.2020.9213 32968442 PMC7500050

